# Comprehensive Cleft Care in a Nine-Year-Old With Unilateral Cleft Lip and Palate Using Coffin Spring Expansion and Surgical Intervention

**DOI:** 10.7759/cureus.64939

**Published:** 2024-07-19

**Authors:** Japneet Kaiser, Ranjit Kamble, Srushti Atole

**Affiliations:** 1 Department of Orthodontics, Sharad Pawar Dental College and Hospital, Datta Meghe Institute of Higher Education and Research, Wardha, IND

**Keywords:** tongue flap, cleft, clp, fistula closure, expander

## Abstract

Cleft lip and palate (CLP) represent a common congenital defect, which needs a multidisciplinary team approach for satisfactory aesthetic and functional correction. Transverse discrepancies are among the major problems in CLP cases, and maxillary expansion has been used to correct skeletal and dental transverse discrepancies between the mandible and maxilla. During the long period, many types of expansion protocols have been cited in the literature. This report presents the treatment of a nine-year-old patient with unilateral CLP and emphasizes the combination of orthodontic and surgical interventions. The maxillary expansion was achieved by Coffin spring, and then oronasal fistula closure surgery was done for both function and aesthetic purposes.

## Introduction

Cleft lip and palate (CLP) are among the common congenital defect that involves the oral region and facial structures, which create difficulties in feeding, speech, and aesthetics. The management of CLP requires a multidisciplinary team, which corrects the disability in a sequential manner combining orthodontic intervention and surgical procedures. Orthodontic intervention is a crucial part of the treatment of cleft patients [1]. Transverse discrepancies are among the major problems in CLP, and maxillary expansion is indicated to eliminate skeletal and dental transverse discrepancies between the mandible and maxilla [2]. Achieving and maintaining orthodontic expansion is the major aim of orthodontic treatment in cleft patients, followed by eruption of teeth in healthy periodontal tissues and acceptable facial aesthetics [3,4].

This case report presents a nine-year-old patient with unilateral CLP treated with a Coffin spring for expansion of the arch, followed by fistula closure. Unilateral CLP patients often exhibit maxillary hypoplasia due to the disruption of normal maxillary growth. Coffin spring expansion helps in expanding the maxillary arch, promoting more symmetrical growth and development. Adequate expansion of the maxillary arch can provide better access for subsequent surgeries, such as alveolar bone grafting, thereby improving the success rates of these procedures. Pre-surgical maxillary expansion can lead to more favorable outcomes in primary and secondary surgical interventions by ensuring better anatomical positioning and alignment.

A Coffin spring is an omega-shaped (Ω) spring invented by Sir Walter Coffin in 1875. It is made of heavy-gauge wire that crosses the palate and is part of a removable appliance [5]. This type of spring allows the maxillary dental arch to potentially expand or contract. In this particular case, the Coffin spring was utilized to expand the arch, thus, creating more space and favorable alignment for the teeth.

The surgical team closed the oronasal fistula, followed by the expansion. Closure of oronasal fistulas is critical in preventing nasal regurgitation of food and liquids, which can lead to chronic irritation, infections, and social embarrassment. Oronasal fistulas can cause hypernasal speech. Closure surgery significantly improves speech quality by restoring the normal separation between the oral and nasal cavities. Persistent fistulas can serve as a pathway for infections. Surgical closure reduces the risk of chronic infections and associated complications. By closing the fistula, patients can maintain better oral hygiene, which is crucial for overall dental health. Tongue flaps are particularly useful for the healing of big fistulas in palates damaged from prior surgery due to their outstanding vascularity and the significant volume of tissue they supply in cleft palate surgery. This report is intended to elaborate on the successful management of unilateral CLP, putting emphasis on why it is advantageous to have both appropriate surgical strategies and expansion methods such as Coffin spring.

## Case presentation

**Figure 1:**
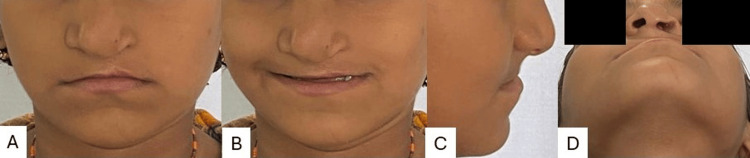
Pre-treatment extraoral images A) Frontal; B) frontal smiling; C) profile; D) worms or basal view

A nine-year-old female patient came to a department with the chief complaint of unpleasant aesthetics because of unilateral cleft lip and palate, irregularly placed teeth, and difficulty in speech. The patient had a history of lip repair surgery at the age of 11 months. There was no significant medical history. During clinical evaluation for extra-oral features, it was found that she presented with a concave profile with a retro-positioned maxilla, flattened nasal tip and depressed nasal dome, and diminished malar prominence (Figure 1).

**Figure 2:**
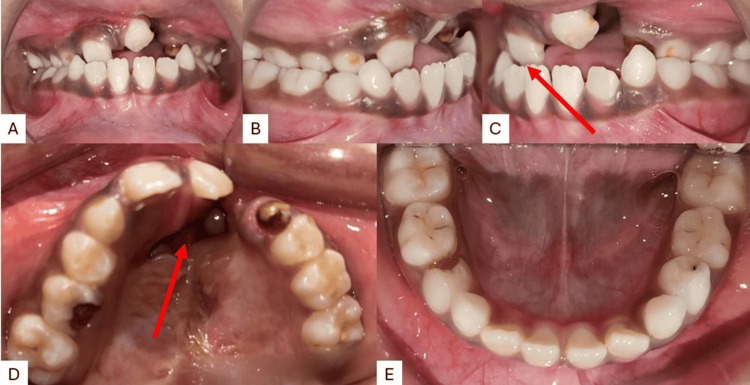
Pre-treatment intraoral images A) Frontal occlusion; B) right occlusion; C) left occlusion; D) maxillary arch; E) mandibular arch

Intra-oral examination revealed a prominent oronasal fistula perforation on the left side with mixed dentition with permanent central incisor in the upper right and left quadrants and deciduous canine and first and second molar in the upper right and left quadrants. Permanent first molars are seen in all four quadrants. The lower arch had all four permanent incisors with deciduous canines and first and second molars in both the lower right and left quadrants. The mandibular arch showed relatively normal condition as compared to the maxilla. Reverse overjet was observed depicting that the maxilla is either retrognathic or retro-positioned (Figure 2).

**Figure 3:**
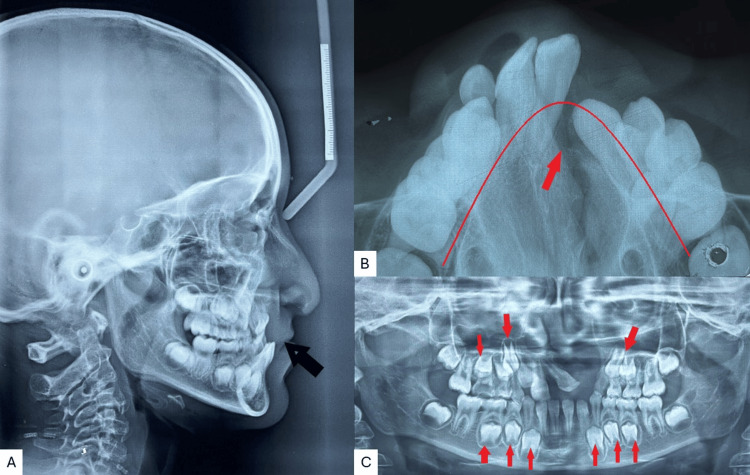
Pre-treatment radiographs A) Lateral cephalogram; B) maxillary occlusal; C) orthopantomogram (OPG)

Radiographic examination and the cephalometric analysis revealed a Class III skeletal pattern, with a retro-positioned maxilla, vertical growth pattern, reclined upper incisors, and normal inclined lower incisors (Figure 3A). The maxillary occlusal radiograph shows a V-shaped arch with a cleft on the left side and constriction in both the anterior and posterior segments (Figure 3B). The orthopantomogram (OPG) examination showed an unerupted upper right lateral incisor, developing tooth buds of the upper right canine, first and second premolars, and a second permanent molar. The upper left quadrant showed the first and second premolars with the permanent second molar, and in the third and fourth quadrant canines, both premolars and second molars were seen (Figure 3C).

The patient was determined to have a Class III skeletal profile and a vertical development pattern based on all clinical investigations and radiographic analysis. The mandible was normal in this instance, but the maxilla's hypoplastic growth gave it a retrognathic appearance with a constriction arch.

Treatment plan and progress

**Figure 4:**
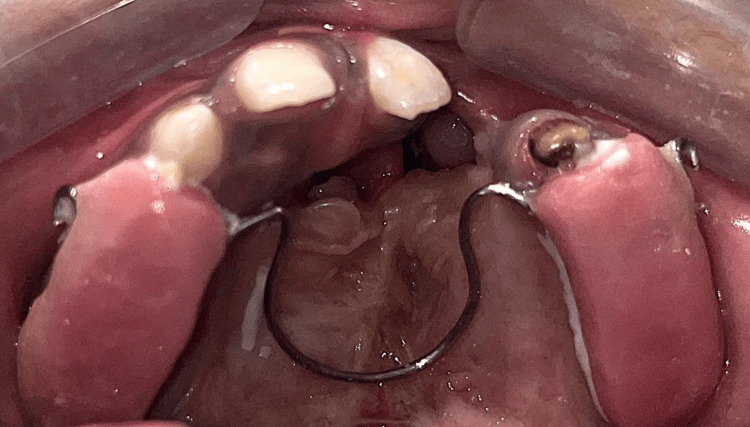
Cementation of the Coffin spring in the maxillary arch Placement of the Coffin spring in the maxillary arch for expansion.

The treatment plan in this instance involved anterior and posterior maxillary expansions, which were followed by fistula closure. The initial phase in the procedure was to fabricate a Coffin expander. The maxillary arch's expander was securely anchored in position (Figure 4) with the help of glass ionomer cement. It was activated in order to obtain a growth of the maxillary arch every four weeks.

**Figure 5:**
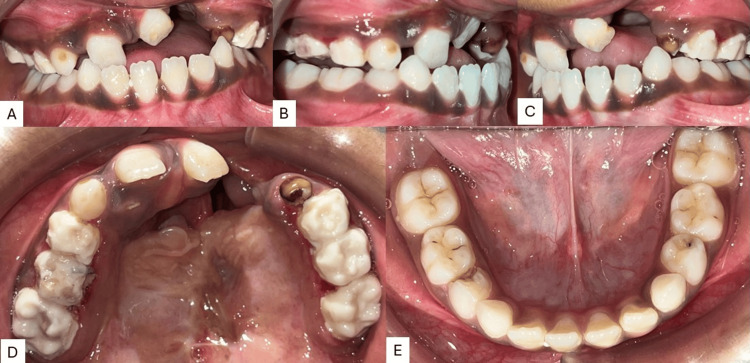
Post-expansion intraoral images A) Frontal occlusion; B) right occlusion; C) left occlusion; D) maxillary arch; E) mandibular arch

Maxillary anterior and posterior enlargement was attained after six months of stimulation (Figure 5). After six months, there was an increase in arch width along with anterior and posterior expansion (Figure 5D).

**Figure 6:**
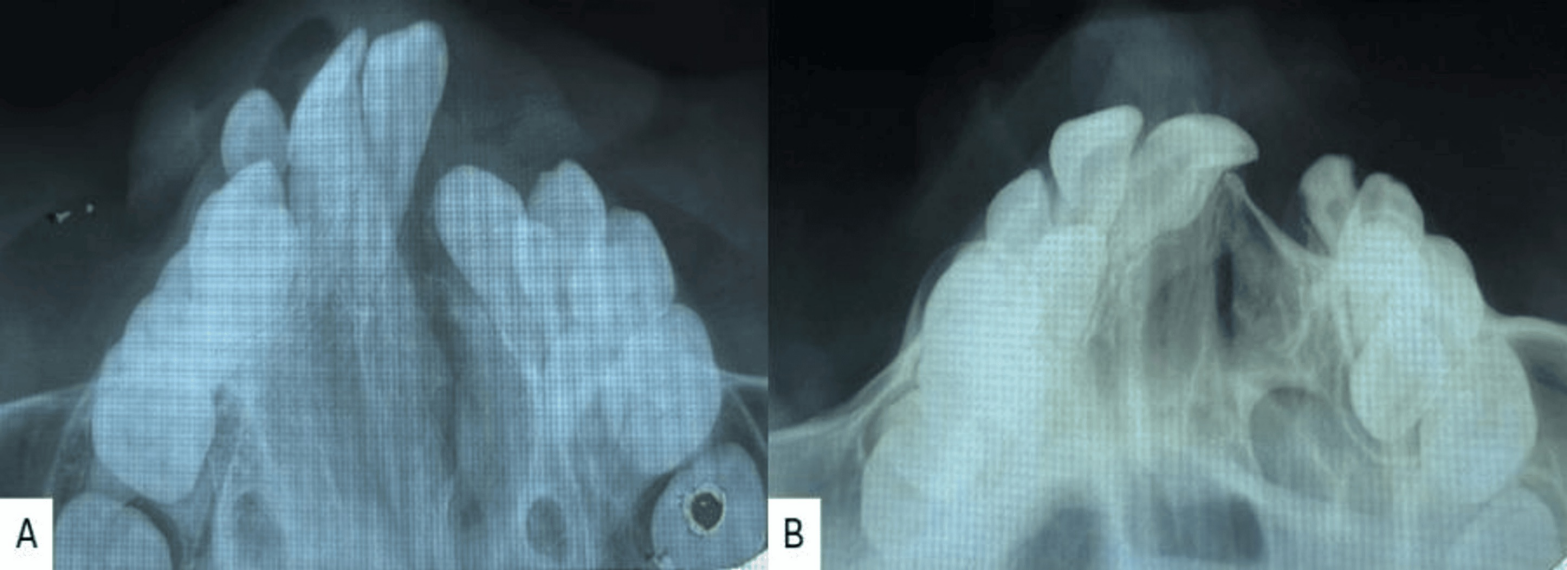
Radiographic comparison of the maxillary arch expansion A) Pre-treatment; B) post-expansion

**Figure 7:**
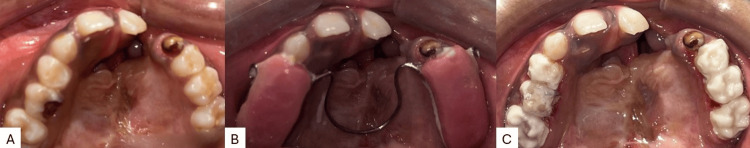
Maxillary arch expansion A) Pre-treatment; B) mid-treatment; C) post-expansion

**Figure 8:**
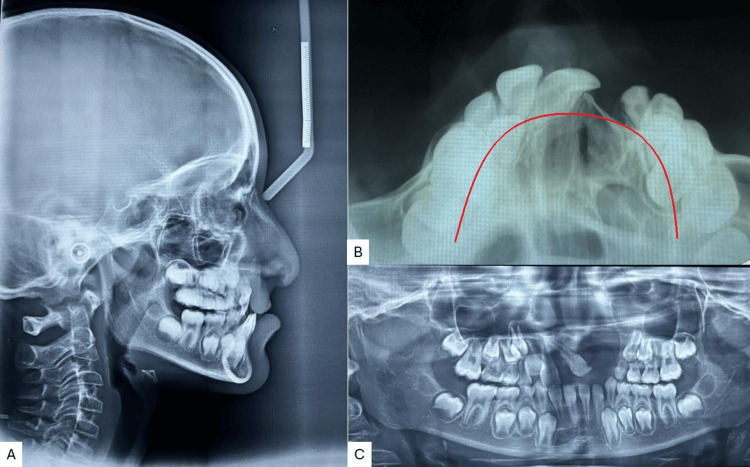
Post-expansion radiographs A) Lateral cephalogram; B) maxillary occlusal radiograph; C) orthopantomogram (OPG)

Both radiographically (Figure 6) and clinically (Figure 7) can identify comparable growth. However, as the modifications were mostly in the transverse plane, the lateral cephalogram and orthopantomogram (Figure 8) do not exhibit any appreciable changes following expansion.

**Figure 9:**
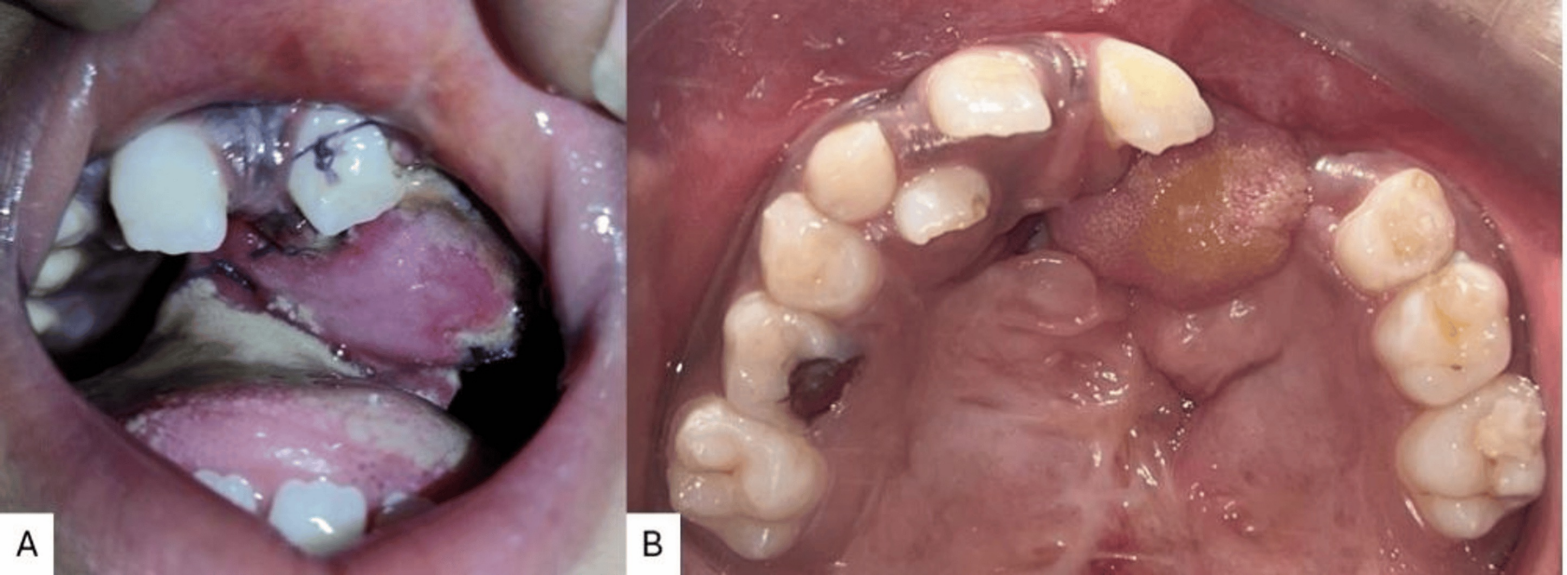
Fistula closure using a tongue flap A) Tongue flap sutured at the fistula region; B) maxillary arch post suture removal

After the expansion was achieved, the patient underwent oronasal fistula closure using a tongue flap (Figure 9), following which the expander was recemented to maintain the expansion correction until the patient underwent secondary alveolar bone grafting. On the follow-up appointment, the patient's sutures were removed, and the flap condition was seen. The flap was seen to have proper blood supply, and good healing was seen. The fistula closure was successful as the patient had no more complaints of oro nasal fistula with fluids passing through her nose on drinking or holding water in her mouth. The bone grafting was not done at the same time as the patient’s guardians did not consent to the procedure.

## Discussion

The treatment of CLP presents a unique set of challenges, requiring a well-coordinated, multidisciplinary approach [6]. This case report highlights the successful management of a nine-year-old patient with unilateral CLP, emphasizing the importance of combining orthodontic expansion with surgical interventions.

One of the key aspects of this treatment was the use of the Coffin spring appliance for maxillary expansion. Sir Walter Coffin (1875) invented the Coffin spring. The appliance comprises an omega-shaped wire put in the mid-palatal area and an Adam’s clasp in the first premolars and first molars on both sides. The appliance is made from stainless steel wire with a diameter of 1.2 mm that is embedded in an acrylic baseplate. The appliance is mainly aimed at causing dental and maxillary changes in instances of one-sided crossbite or two-sided crossbite, cases requiring lateral expansion, anteroposterior expansion cases, and when there is less than a 3-mm gap needed. In the mixed dentition period, a certain amount of skeletal changes can also result if proper retention protocol is followed [7]. The upper jaw was gradually expanded by the Coffin spring, which can put mild but efficient force to create it. This led to enough space for teeth to correctly form amongst the bone and prepare for future surgeries. In order to manage the patient's maxillary constriction and prepare the patient for surgery, the appliance proved to be a flexible and useful tool.

Alveolar bone grafting and fistula closure were two crucial surgical techniques involved in the repair. The patient's speaking and eating abilities improved when the oronasal fistula, a typical complication in CLP patients, was successfully closed. This prevented food and liquids from migrating between the mouth and nose. A combination of an endoscopic and per-oral BFP flap approach, BFP flap, pedicled buccal fat pad, modified submucosal connective tissue flap, distant flaps, autogenous bone grafts, allogenous, synthetic materials/metals, and other techniques can be used to close defects larger than 5 mm. Both soft tissue and bone closure of OAF are ideally achieved with the Bio-Gide®-Bio-Oss®^TM^ sandwich approach (Geistlich Biomaterials, Wolhusen, Switzerland) [8]. After expansion, an alveolar bone graft was necessary to stabilize the dental arch and lay the basis for future dental growth. Additionally, by supporting the emergence of permanent teeth, this treatment helped maintain the maxilla's general structural integrity.

Achieving these results required the orthodontic and surgical teams to work together. A thorough and all-encompassing approach to the patient's care resulted from effective planning and communication, which made sure that each stage of treatment complimented the others. The above scenario emphasizes the need for a customized treatment strategy that takes into account the unique requirements of CLP patients and combines surgical and orthodontic methods to provide the best possible functional and esthetic results.

To monitor the patient's growth and development and make sure the treatment's effects are maintained, long-term follow-up will be essential. As the patient ages, additional dental and orthodontic procedures might be needed, but for now, this combination approach has put the patient on a path to better oral health and general well-being.

## Conclusions

The management of unilateral CLP using Coffin spring expansion combined with oronasal fistula closure surgery has shown promising results. This case report highlights the effectiveness of this approach in achieving functional and aesthetic improvements. The Coffin spring expansion facilitated the alignment of the maxillary segments, promoting better dental arch form and improving occlusion. The subsequent oronasal fistula closure surgery successfully addressed the communication between the oral and nasal cavities, significantly reducing the risk of recurrent infections and improving speech outcomes.

The patient's post-operative follow-up demonstrated notable improvements in both structural integrity and overall quality of life. This combined approach not only provided a stable and symmetrical facial appearance but also enhanced oral function, speech clarity, and psychological well-being. The integration of these techniques underscores the importance of a comprehensive and multidisciplinary approach in the treatment of unilateral CLP.
